# Giant sinus tract formation following percutaneous renal biopsy in a patient with systemic lupus erythematosus: A case report

**DOI:** 10.1097/MD.0000000000046780

**Published:** 2026-01-09

**Authors:** Xiaoling Yan, Xiaokui Fu, Weiren Li

**Affiliations:** a Department of Burn and Plastic Surgery, Guizhou Provincial People’s Hospital, Guiyang, China; b Department of Anesthesiology, Affiliated Hospital of Guizhou Medical University, Guiyang, China; c Department of Burn and Plastic Surgery, Affiliated Hospital of Guizhou Medical University, Guiyang, China.

**Keywords:** case report, giant sinus tract, magnetic resonance imaging, percutaneous renal biopsy, systemic lupus erythematosus

## Abstract

**Rationale:**

Although percutaneous renal biopsy is generally considered safe, it can result in complications such as sinus tract formation, particularly in immunocompromised patients. In systemic lupus erythematosus (SLE), prolonged immunosuppression impairs tissue repair, complicating management. No standardized treatment exists for post-biopsy sinus tracts in SLE, and conventional approaches often yield poor results. A case of a giant sinus tract after renal biopsy in an SLE patient, successfully managed with multidisciplinary therapy, offering a potential reference for similar complex cases.

**Patient concerns:**

A 38-year-old woman with an 8-year history of SLE and lupus nephritis was admitted due to persistent redness and exudation at the renal biopsy site for 5 months. Prior treatments at an outside hospital, including intravenous antibiotics, abscess incision and drainage, and routine dressing changes, were ineffective, and her symptoms progressively worsened. Imaging revealed an irregular sinus tract measuring approximately 10 cm × 9 cm in the right lumbar region.

**Diagnoses:**

Giant sinus tract formation following percutaneous renal biopsy.

**Interventions:**

Standard immunosuppressive therapy was initiated to control SLE. On day 5, the patient underwent debridement combined with negative pressure wound therapy with instillation using hydrogen peroxide. Medications were adjusted with the support of the nephrology department. On day 13, radical excision of the sinus tract, further debridement, and skin flap grafting were performed. Dual-mode negative pressure drainage was applied to accelerate healing, and systemic antibiotics were administered based on culture and sensitivity results.

**Outcomes:**

By day 40 of hospitalization, the wound was completely epithelialized, without redness or discharge. The patient achieved complete clinical healing and remained recurrence-free during a 4-year follow-up.

**Lessons:**

Despite the relatively low post-procedural infection rate in SLE patients, clinicians should remain vigilant for complications such as sinus tract formation at the biopsy site. Early imaging is essential for timely diagnosis and preventing missed diagnoses. In this case, a combination of immunomodulation, negative pressure wound instillation therapy, and local skin flap reconstruction, along with dual-mode negative pressure drainage, demonstrated high debridement efficiency, minimal trauma, improved healing, better prognosis, and a low recurrence rate.

## 1. Introduction

A sinus tract is a blind-ended channel formed by tissue ulceration that extends from a deep lesion to the skin surface. It typically represents a secondary manifestation of infection, inflammation, tumors, or surgical complications. Although not a disease entity in itself, it is characterized by insidious onset, prolonged treatment duration, and a high risk of recurrence, all of which can significantly impair patient prognosis.^[1–3]^

Although the safety of percutaneous renal biopsy is widely acknowledged, there remains a risk of complications such as sinus tract formation, especially in immunocompromised individuals. Patients with systemic lupus erythematosus (SLE) often experience impaired tissue repair due to prolonged immunosuppressive therapy, rendering the management of such complications more challenging.^[4,5]^ Currently, there is no standardized treatment protocol for post-biopsy sinus tracts in SLE patients, and conventional interventions often yield unsatisfactory outcomes. A case of a giant sinus tract following percutaneous renal biopsy in a patient with SLE. The wound was successfully healed through a comprehensive treatment approach combining immunomodulation, staged surgical interventions, and negative pressure wound therapy. This case provides a valuable reference for the management of similarly complex presentations.

## 2. Case description

A 38-year-old female patient was admitted to the Department of Burn and Plastic Surgery, Affiliated Hospital of Guizhou Medical University, on October 20, 2018, due to persistent nonhealing of the percutaneous right renal biopsy site and recurrent exudation for 5 months. In May 2018, she underwent a percutaneous right renal biopsy at an outside hospital. Two weeks after the procedure, she developed localized redness, pain, and fever (up to 39°C) at the puncture site. Empirical systemic antibiotic therapy (specific agents unknown) and 20% magnesium sulfate wet compresses were administered, resulting in partial symptom relief. One month postoperatively, the erythema extended to the right lower back, accompanied by skin ulceration and fluctuance. Incision and drainage of the abscess were performed under local anesthesia at the referring hospital, which alleviated the redness and swelling. However, the drainage site failed to heal, with persistent light yellow exudate of 30 to 40 mL/d. Despite prolonged wound care, no significant improvement was observed, and the patient was referred to our hospital for further management.

### 2.1. Relevant examinations

#### 2.1.1. Physical examination

**Figure 1.**
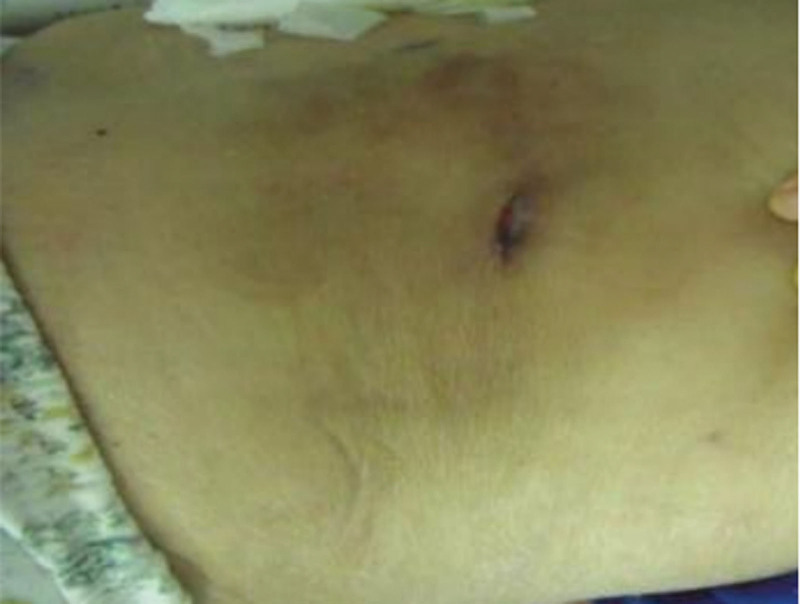
Sinus opening located at the lateral lumbar region, with no surrounding redness or swelling.

Multiple scattered erythematous patches with surface scaling were observed on the patient’s lower limbs, right forearm, and trunk. A characteristic ulcerative lesion measuring 2.0 cm × 1.0 cm was noted on the right lower back (Fig. 1). Probing revealed a sinus tract with a maximum depth of 7 cm. The wound exuded a clear, light yellow fluid, with mild tenderness on palpation and no obvious surrounding erythema or swelling.

#### 2.1.2. Laboratory tests

Hematological examination revealed leukocytosis (11.71 × 10⁹/L), anemia (hemoglobin 85.00 g/L), and a normal platelet count (158.00 × 10⁹/L). Coagulation tests showed prolonged prothrombin time (20.4 seconds), activated partial thromboplastin time (41.4 seconds), thrombin time (24.4 seconds), and elevated fibrinogen levels (6.46 g/L). Biochemistry results indicated hypoproteinemia (albumin 25.30 g/L) and hypokalemia (3.38 mmol/L). Urinalysis revealed marked proteinuria (+++).

#### 2.1.3. Imaging evaluation

**Figure 2.**
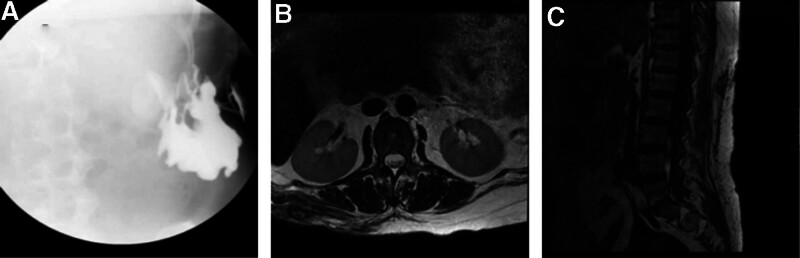
(A) X-ray imaging revealed an irregular sinus tract in the right lumbar region, measuring approximately 10.0 × 9.0 cm. (B, C) MRI showed a patchy, heterogeneous signal at the L2 level, with a maximum cross-sectional area of about 7.8 × 6.3 cm. MRI = magnetic resonance imaging.

Lumbar spine MRI revealed a patchy abnormal signal at the L2 level of the right lumbar region, with a maximum cross-sectional area of approximately 7.8 cm × 6.3 cm. Sinus tract radiography confirmed an irregular sinus tract in the right lumbar region, measuring approximately 10.0 cm × 9.0 cm. The right kidney was not visualized (Fig. 2A–C).

#### 2.1.4. Microbiological analysis

Culture of sinus tract secretions identified *Leclercia adecarboxylata*. Drug susceptibility testing indicated sensitivity to imipenem and cefoperazone–sulbactam.

### 2.2. Diagnosis

The final diagnosis was a giant sinus tract formation following percutaneous renal biopsy in a patient with SLE.

### 2.3. Treatment

#### 2.3.1. Immunosuppressive therapy

Upon admission, the patient was initiated on an immunosuppressive regimen consisting of intravenous methylprednisolone (40 mg/d), oral mycophenolate mofetil (500 mg twice daily), and oral hydroxychloroquine (200 mg twice daily).

#### 2.3.2. First stage surgical intervention

**Figure 3.**
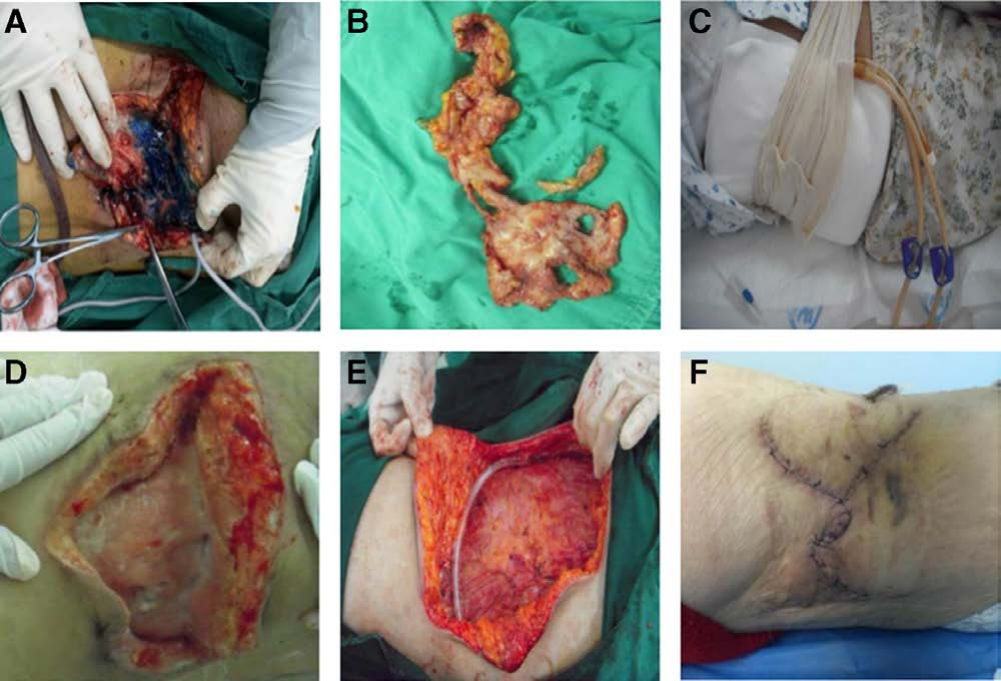
(A) The sinus tract was delineated intraoperatively using methylene blue staining; the cavity measured approximately 16.0 cm × 10.0 cm. (B) Excised necrotic tissue. (C) Application of negative pressure wound therapy (NPWT) over the wound bed, secured with an elastic compression bandage. (D) Wound bed appeared cleaner with reduced exudation after NPWT removal. (E) Radical excision of the sinus tract extending to the muscularis resulted in a full-thickness skin defect measuring approximately 20.0 cm × 15.0 cm. (F) Postoperative day 31: the surgical site showed no erythema, the incision was well approximated, and only minimal exudate was observed at the drainage tube opening. NPWT = negative pressure wound therapy.

On hospital day 5, the patient underwent debridement of the lumbosacral sinus tract combined with negative pressure wound therapy with instillation (NPWTi). Intraoperatively, hydrogen peroxide and saline were used repeatedly for irrigation, and methylene blue was injected into the sinus tract to delineate the extent of the lesion. Surgical exploration revealed a wound cavity extending to the deep fascia, measuring approximately 16 cm × 10 cm. Postoperatively, intravenous cefoperazone–sulbactam (2 g twice daily) was administered based on drug sensitivity results (Fig. 3A–C).

#### 2.3.3. Treatment adjustment and second stage surgery

Given the effective infection control from the first stage, the NPWTi device was removed on hospital day 14. Wound assessment revealed a clean wound bed (Fig. 3D). Steroid therapy was then adjusted to oral prednisone (45 mg/d). On day 26, an extended debridement procedure was performed, involving complete excision of the sinus tract invading the muscular layer. The procedure resulted in a full-thickness skin defect measuring approximately 20 cm × 15 cm, which was repaired with a local skin flap (Fig. 3E). Dual negative pressure drainage devices were placed, with pressure settings ranging from −200 to −400 mm Hg.

### 2.4. Treatment outcomes and follow-up

**Figure 4.**
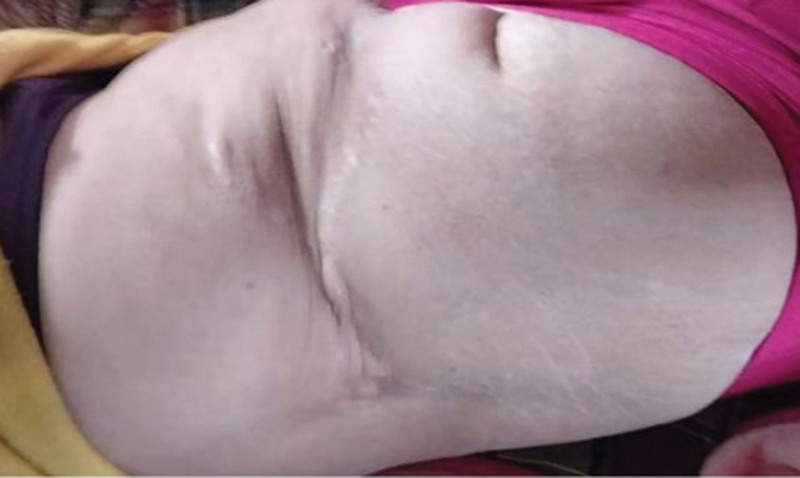
At the 4-year postoperative follow-up, the scar was flat, soft, and closely matched the surrounding skin tone.

On hospital day 31, the negative pressure drainage devices were removed, with good flap viability and no erythema around the wound margins (Fig. 3F). By day 38, the drainage volume had decreased to <10 mL in 24 hours, allowing for removal of the drains and suture removal. The patient was discharged on hospital day 40 after complete recovery. During a 4-year follow-up period, no recurrence of the sinus tract was observed. The surgical site demonstrated a flat, soft scar with normal pigmentation and satisfactory appearance (Fig. 4).

## 3. Discussion

Patients with SLE often experience significantly impaired tissue repair due to prolonged immunosuppressive therapy, posing unique challenges in the management of chronic, nonhealing wounds.^[6]^ Out of concern for serious complications such as hemorrhage, infection, and poor tissue regeneration, clinicians often favor conservative treatment strategies.^[7]^ However, clinical observations in this case suggest that such approaches are frequently ineffective for established giant sinus tracts. A closer examination of the reasons for the failure of conventional treatments points to a complex interplay of multiple pathological factors. Anatomically, sinus tracts often have multiple branches and tortuous, narrow channels that hinder adequate coverage of the wound base with conventional dressings, directly limiting the effectiveness of local wound care.^[8]^ Microbiologically, *L adecarboxylata* isolated from the sinus secretions has a strong capacity for biofilm formation, promoting persistent bacterial colonization and deep tissue invasion. This may explain the failure to achieve healing despite systemic antibiotic therapy and drainage.^[9]^ More importantly, SLE patients often exhibit histopathological changes such as cutaneous small-vessel vasculitis and mucin deposition.^[10]^ These are compounded by microcirculatory disturbances due to long-term corticosteroid use and systemic metabolic imbalances – such as anemia and hypoproteinemia – all of which contribute to a multifactorial basis for impaired wound healing.^[10–15]^

Based on the above understanding, a systematic treatment strategy was developed. Key measures in the early phase of treatment included maintaining baseline immunosuppressive therapy while dynamically adjusting the regimen in collaboration with the nephrology team to achieve a balance between inflammation control and tissue repair.^[16]^ Imaging modalities such as sinus tract radiography and MRI were employed to accurately assess the anatomical course of the sinus and its relationship to adjacent structures, providing a foundation for surgical planning.^[17]^ A staged surgical approach was adopted. The first stage focused on infection control. Vacuum sealing drainage combined with hydrogen peroxide irrigation was applied to effectively reduce the local bacterial load, disrupt biofilm structure, and alleviate inflammation, thereby creating favorable conditions for subsequent wound reconstruction.^[18]^ Intraoperatively, the extent and depth of the sinus tract as well as its histopathological features were further delineated, and special pathogen infections were excluded through pathological examination.^[19]^ The second stage involved radical debridement followed by local skin flap reconstruction. Local skin flaps, which carry their own vascular pedicle, provide stable perfusion and strong resistance to infection with minimal trauma, making them an ideal option for immunocompromised patients. Postoperatively, a vacuum sealing drainage device was placed over the skin flap to provide continuous negative pressure. This approach helped reduce wound tension, minimized the risk of hematoma and seroma formation, lowered the incidence of postoperative infection and wound dehiscence, and also contributed to reduced scar formation.^[20,21]^ Notably, cefoperazone–sulbactam was selected for targeted antimicrobial therapy based on drug susceptibility testing, embodying the principles of precision medicine. This technique design was particularly useful for addressing such complex, infection-related sinus tracts because each component of the approach targeted a key pathogenic mechanism. Specifically, the early application of NPWTi enhanced mechanical debridement and promoted granulation tissue formation by maintaining continuous drainage and local perfusion, thus improving tissue oxygenation in the hypoperfused area. Meanwhile, the integration of preoperative multimodal imaging (MRI, ultrasound, and radiography) allowed accurate delineation of the sinus tract’s course and adjacent anatomical structures, ensuring complete excision while preserving viable tissue. The sequential combination of infection control and flap reconstruction optimized both microbial clearance and wound closure in immunocompromised patients, ultimately shortening healing time and reducing recurrence risk.

Furthermore, in-depth analysis of the diagnostic and therapeutic process in this case revealed that early missed diagnosis was a key factor contributing to disease progression. This issue can be primarily attributed to 2 factors: first, the patient’s early clinical manifestations were atypical, with only a small superficial skin defect at the sinus opening, which was easily overlooked; second, sinus tract formation following renal biopsy is a rare complication and is thus underrecognized in clinical practice. In fact, a systematic clinical assessment could have identified a characteristic early triad of sinus tract development: erythema around the sinus opening, persistent exudation, and fever.^[22]^ More importantly, modern imaging technologies – including ultrasonography, sinus tract radiography, computed tomography angiography, and MRI – can accurately delineate the morphology and extent of the subcutaneous cavity^[23]^, while microbiological and histopathological analyses provide robust evidence for identifying infectious etiologies. From the perspective of preventing post-biopsy infectious complications, a structured intervention pathway for high-risk patients based on “risk assessment–precision management–early monitoring” is proposed – preoperative risk assessment: in patients with active disease, prolonged high-dose corticosteroid use, or malnutrition, the necessity of renal biopsy should be rigorously evaluated. Individualized procedural plans should be established, and image-guided precision techniques should be prioritized to reduce the risk of potential complications.^[24]^ Intraoperative optimization: under strict aseptic conditions, preference should be given to minimally invasive, image-guided biopsy techniques (such as real-time ultrasound guidance or image fusion navigation) to minimize tissue trauma and avoid repeat puncture.^[25]^ Postoperative early monitoring: high-resolution imaging modalities (e.g., high-frequency ultrasound, X-ray, computerized tomography angiography, and MRI) should be actively employed for surveillance. Clinical manifestations, physical signs, and microbiological findings should be closely integrated to detect early signs of infection or sinus formation, thereby enabling timely diagnosis and intervention.^[26]^

The therapeutic experience from this case offers important insights for clinical practice. First, in treating biofilm-associated infections, it is essential to consider not only the antibiotic susceptibility of the pathogen but also the ability of the selected agent to penetrate biofilms.^[27]^ Adjunctive physical and chemical methods – such as negative pressure wound therapy combined with irrigation systems and the local application of antimicrobial agents – can enhance biofilm disruption,^[28,29]^ while surgical debridement remains the cornerstone of treatment. The preoperative integration of multiple imaging techniques (including MRI, ultrasound, and radiography) can accurately delineate the extent of the lesion, guide preoperative diagnosis, inform incision planning, and optimize surgical approach design. This multimodal imaging strategy improves surgical precision by enabling thorough removal of necrotic and biofilm-colonized tissues while preserving as much healthy tissue as possible.^[30–33]^ Additionally, intraoperative injection of methylene blue can aid in demarcating lesion boundaries, thereby avoiding unnecessary tissue damage from excessive debridement.^[34]^ Second, in immunocompromised patients with concurrent anemia and hypoproteinemia – conditions that markedly impair wound healing and infection control – choosing an appropriate reconstructive approach becomes crucial, particularly when managing large sinus tracts as in the present case. A local skin flap technique, which is relatively simple to perform, effective, minimally invasive, and not highly dependent on vascular status, is especially advantageous in such settings.^[35]^ In summary, a comprehensive, multidisciplinary approach combining detailed assessment, targeted infection control, and tailored tissue reconstruction forms an integrated treatment system for managing complex, refractory sinus tracts.

## 4. Conclusion

Although favorable clinical outcomes were achieved in this case, the prolonged treatment duration and considerable economic burden warrant further clinical attention. Moreover, as a single-case report, the generalizability of the treatment strategy remains to be confirmed. Future research should focus on 2 key directions. First, optimizing immunological microenvironment regulation. On the basis of effective control of SLE disease activity, it is crucial to explore improved immunosuppressive regimens or combination therapies incorporating pro-healing factors to enhance tissue repair and shorten wound healing time. Second, evaluating the clinical value of minimally invasive endoscopic techniques for managing chronic and complex sinus tracts. These approaches, characterized by minimal trauma, procedural flexibility, and relatively low cost, are already widely used in other surgical fields and warrant systematic evaluation of their efficacy and safety through multicenter, prospective clinical studies. By continuously refining comprehensive treatment strategies and integrating innovative interventions, it may be possible to improve therapeutic efficiency, reduce healthcare burden, and further enhance the prognosis of refractory wounds associated with immunosuppression.

## Acknowledgments

We thank the patient for participating in this case report.

## Author contributions

**Data curation:** Xiaokui Fu.

**Formal analysis:** Xiaokui Fu.

**Supervision:** Weiren Li.

**Writing – original draft:** Xiaoling Yan.

**Writing – review & editing:** Xiaoling Yan.
